# Impact of Immediate Dentin Sealing With Various Universal Adhesives on Shear Bond Strength of Dual‐Cure Resin Cement

**DOI:** 10.1002/cre2.70186

**Published:** 2025-08-12

**Authors:** Malin Janson, Li Sun, Anja Liebermann, Christoph Matthias Schoppmeier

**Affiliations:** ^1^ Department of Prosthetic Dentistry University of Cologne, Faculty of Medicine and University Hospital Cologne Cologne Germany; ^2^ Polyclinic for Operative Dentistry and Periodontology University of Cologne, Faculty of Medicine and University Hospital Cologne Cologne Germany

**Keywords:** 10‐MDP, immediate dentin sealing, resin cement, shear bond strength, thermocycling, universal adhesive

## Abstract

**Objectives:**

This study evaluated the effect of universal adhesives (UAs) applied as Immediate Dentin Sealing (IDS) on the shear bond strength (SBS) of dual‐cure resin cement, both immediately and after thermocycling aging.

**Material and Methods:**

A total of 180 bovine incisors were prepared and randomly assigned to six groups (*n* = 15): NOC (control), Clearfil Universal Bond Quick (CUBQ), G‐Premio Bond (GPB), Peak Universal Bond (PUB), Adhese Universal (AU), and Scotchbond Universal Plus (SBUP). Specimens were cemented with dual‐cure resin cement (Panavia V5) and subjected to SBS testing at 24 h and after thermocycling (10,000 cycles, 5°C–55°C). SBS was measured using a universal testing machine, and failure modes were assessed microscopically. Statistical analysis was conducted using a Generalized Linear Model with Bonferroni correction (*p* < 0.05).

**Results:**

Significant differences were found among UAs (*p* < 0.001) and after aging (*p* < 0.001). SBUP and GPB had the highest SBS before and after thermocycling, while NOC had the lowest. Thermocycling reduced SBS in all groups, with CUBQ and AU showing the largest declines. Failure mode analysis showed predominantly adhesive failures in NOC, while IDS groups had more cohesive and mixed failures.

**Conclusion:**

IDS technique with universal adhesives significantly enhances bond strength to dentin compared to conventional cementation. SBUP and GPB showed superior bonding performance, likely due to their monomer formulations containing 10‐MDP and acetone, which are known to improve chemical adhesion to dentin and promote effective resin infiltration. Thermocycling led to a reduction in SBS across all groups, highlighting the impact of aging on adhesive durability, underscoring the importance of selecting UAs with lasting adhesive strength for long‐term bonding.

## 1 Introduction

Indirect restorations, such as veneers and partial crowns fabricated from dental ceramics and polymer‐based materials, are gaining increasing popularity in modern restorative dentistry. Although these restorations typically necessitate multiple clinical sessions and incur higher costs compared to direct composite restorations, they offer several distinct advantages. These include precise contact point adaptation, optimal anatomical contouring, and controlled marginal integrity compared to composite restorations. Furthermore, an idealized morphology and optimal interproximal contact can be achieved, thereby enhancing functional outcomes (Samartzi et al. 2021). Additionally, in cases of deep subgingival preparation margins in dentin, indirect restorations allow for a controlled elevation of the cavity floor (Samartzi et al. 2021).

However, preparation for indirect restorations often necessitates substantial removal of tooth structure to achieve adequate material thickness, which increases the risk of dentin tubule exposure. Given the high permeability of dentin immediately after preparation, effective dentin management is critical at this stage (Kimyai et al. 2023). In conventional indirect restorations, exposed dentin serves as a potential entry point for bacterial microleakage and is further subjected to mechanical and chemical stressors during impression‐taking procedures and contamination from temporary cement. These factors can compromise the long‐term bond strength of the final restoration and may induce pulpal symptoms (Kimyai et al. 2023).

The conventional approach, termed delayed dentin sealing (DDS), involves sealing the dentinal tubules only at the final bonding stage (Samartzi et al. 2021). In contrast, the Immediate Dentin Sealing (IDS) technique entails the application of an adhesive system or dentin bonding agent immediately after tooth preparation, before impression‐taking and provisionalization. This method prevents dentin contamination and hypersensitivity while simultaneously enhancing the adhesion of indirect restorations to dentin (Alghauli et al. 2024). The longevity and clinical success of indirect restorations are highly dependent on the quality of the adhesive bond between enamel, dentin, and the restoration surface (Krishnan et al. 2025; Portella et al. 2024). Previous studies suggest that IDS following tooth preparation can significantly improve the shear bond strength (SBS) of resin cement, particularly when large dentin areas are involved (Magne 2005).

In clinical practice, self‐etch dentin bonding agents are frequently preferred due to their simplified application and reduced technique sensitivity. Despite this, their mechanical durability remains inferior compared to other adhesive systems. A potential enhancement of bond strength in self‐etch adhesives is achieved through the “enhanced IDS” approach, wherein a layer of flowable composite resin is applied over the sealed dentin surface. This technique poses challenges such as adhesive pooling and insufficient polymerization (Antoniou et al. 2024). To mitigate these issues, the IDS technique employs adhesive systems that ensure a uniform layer thickness, radiopacity, and superior bond strength (Hardan et al. 2022).

The introduction of new‐generation universal adhesives (UAs), which adhere to ceramics, indirect composite resins, and metal alloys through 10‐methacryloyloxydecyl dihydrogen phosphate (10‐MDP), has simplified bonding procedures (Kimyai et al. 2023). Additionally, these adhesives allow versatile application modes, including etch‐and‐rinse, self‐etch, and selective‐etch approaches. Recent data suggests that the bonding performance of universal adhesives to dentin is comparable regardless of the application mode, as both etch‐and‐rinse and self‐etch techniques achieve effective sealing of dentinal tubules and similar reductions in dentin permeability (Kijsamanmith et al. 2025). Nevertheless, the bond strength of UAs compared to conventional adhesive systems remains a subject of debate in the literature. While some studies report improved bonding performance, others find no significant differences (Lin et al. 2025; Fazlioglu et al. 2025; Samimi et al. 2024). Given the unique chemical formulations of UAs compared to earlier generations, their effectiveness in conjunction with the IDS technique remains insufficiently investigated.

Despite the widespread clinical use of universal adhesives for Immediate Dentin Sealing (IDS), a direct comparison of the bonding effectiveness of currently available formulations within a standardized protocol using Panavia V5 cement has not yet been reported. This study addresses this gap by systematically evaluating five widely used universal adhesives under consistent application and aging conditions.

This study aimed to evaluate the effect of various UAs used for IDS on the SBS of dual‐cure resin cement, with and without artificial aging.

The following null hypotheses were tested:

I. There is no significant difference in the SBS of dual‐cure resin cement to dentin among the investigated universal adhesives for IDS.
II. There is no significant difference in bond strength between restorations cemented with IDS versus those cemented conventionally without prior dentin sealing.

## 2 Methods

### 2.1 Study Sample

The sample size calculation was conducted using the G*Power software (version 3.1, Heinrich Heine University Düsseldorf, Germany), based on the expected effect size between groups, in accordance with previous literature (Rigos et al. 2023). For the SBS test, a minimum of 15 specimens per group was required to achieve a medium effect size (*d* = 0.50) with 95% statistical power and a 5% Type I error rate.

**Figure 1:**
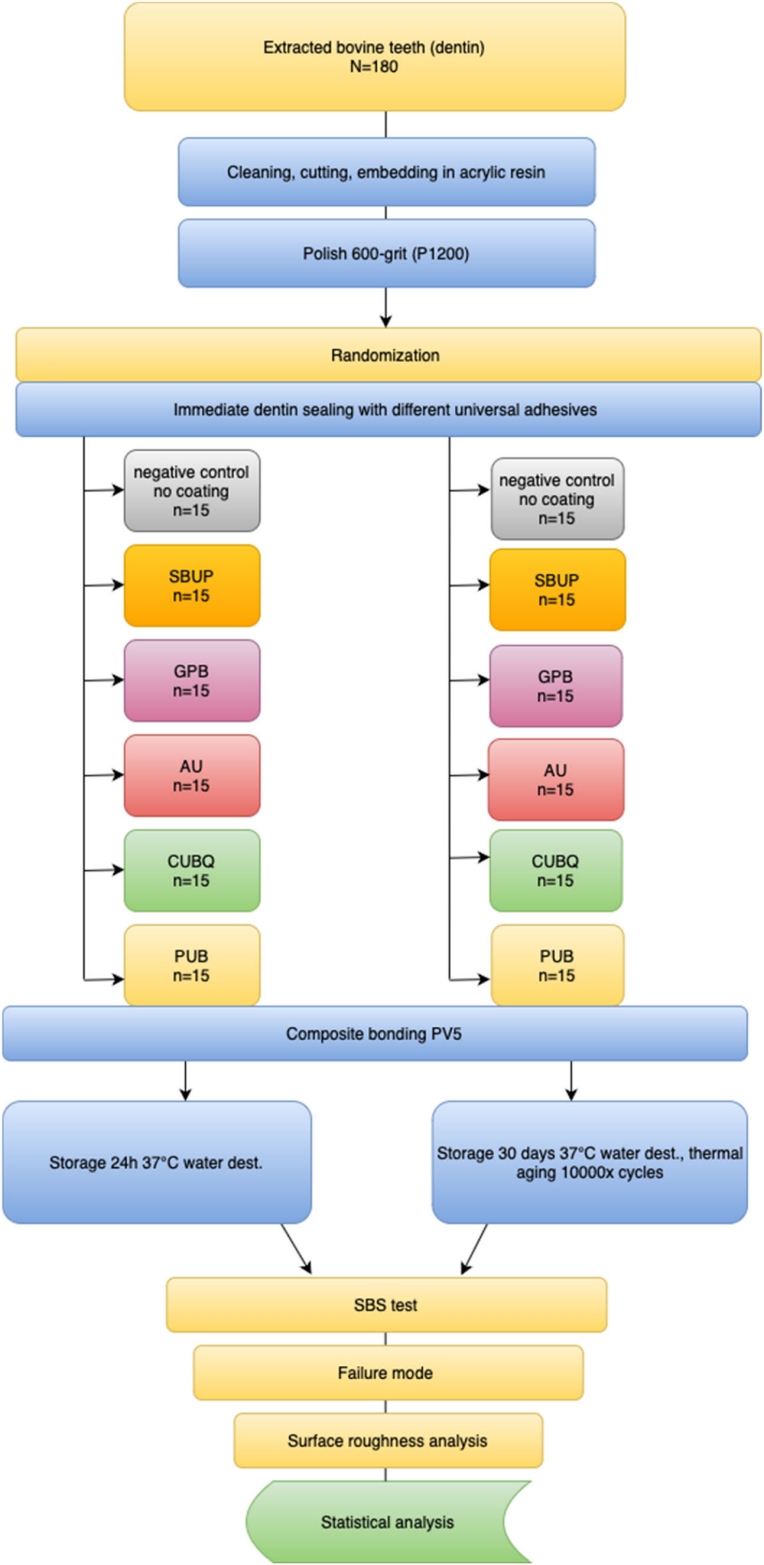
Study flow chart.

A total of 180 caries‐free, intact bovine incisors were extracted from mandibles and stored in a 0.1% thymol solution for 2 weeks to inhibit bacterial growth and prevent dehydration until the experimental procedures. The roots and pulp tissue were removed, and the crowns were thoroughly cleaned using ultrasonic scaling, manual instrumentation with a scaler, and pumice polishing, followed by rinsing with distilled water. The incisors were sectioned, and the enamel was completely removed using a diamond saw under constant water irrigation, exposing the underlying dentin (Secotom‐50, Struers, Ballerup, Denmark; Isomet 1000, Buehler, Lake Bluff, IL, USA). The dentin surfaces were examined under 40× magnification to ensure complete enamel removal and structural integrity of the dentin. Each dentin specimen was then embedded in chemically activated acrylic resin (Palapress Vario, Kulzer GmbH, Hanau, Germany). To standardize the smear layer formation, the dentin surfaces were polished with 600‐grit silicon carbide (SiC) paper under running water (SiC Foil, Struers, Ballerup, Denmark). Following preparation, the specimens were sequentially numbered and randomly assigned to one of six experimental groups, based on the adhesive application protocol (*n* = 15) (Figure 1).

Group 1: NOC, negative control, no IDS‐treatment was applied.

Group 2: SBUP, E&R‐universal adhesive system, Scotchbond Universal Plus, 3M.

Group 3: GPB, E&R‐universal adhesive system, G‐Premio Bond, GC Dental.

Group 4: AU, E&R‐universal adhesive system, Adhese Universal, Ivoclar Vivadent.

Group 5: CUBQ, E&R‐universal adhesive system, Clearfil Universal Bond Quick, Kuraray Noritake.

Group 6: PUB, E&R‐universal adhesive system, Peak Universal Bond, Ultradent Products.

### 2.2 Dentin Preparation

The dentin surfaces were etched with 37% phosphoric acid (K‐Etchant, Kuraray Noritake, Okayama, Japan) for 15 s, rinsed with water for 10 s, and gently dried with cotton pellets. Subsequently, UAs were applied following the Immediate Dentin Sealing (IDS) protocol (Magne 2005), according to the specific application instructions for each adhesive system. The materials used in this study, including compositions, batch numbers, and application protocols, are summarized in Table 1. Following application, the bonding agents were polymerized (1200 mW/cm²/20 s) using an LED light‐curing unit (Bluephase Style, Ivoclar Vivadent, Schaan, Liechtenstein), maintaining a perpendicular angle to the dentin surface (Magne 2005). To prevent oxygen inhibition, a layer of glycerin gel (Liquid Strip, Ivoclar Vivadent, Schaan, Liechtenstein) was applied before a final 20‐s light‐curing step, as described by Magne et al (Magne 2005). After polymerization, the glycerin gel was thoroughly rinsed off with water, and the dentin surfaces were gently air‐dried using cotton pellets (Magne 2005). The specimens were stored in distilled water at 37°C for 72 h before cementation to allow for radical decay and optimal adhesion stability. Before cementation, the sealed dentin surfaces were sandblasted with 50 μm aluminum oxide particles (0.2 MPa, 10 s) to enhance bonding performance.

**Table 1:** Brand names, manufacturers, batch numbers, chemical composition, and applications of materials used.

Brand/Manufacturer	Abbreviation	Batch number	Chemical composition	Application
Panavia V5 Kuraray Noritake Dental Inc., Okayama, Japan	PAN	3U0276	Silanated barium glass filler (30%–70%) Hydrophobic aromatic dimethacrylate (10‐30%), Bis‐GMA (5%–15%) Silanated fluoroalminosilicate glass filler (1%–10%), Hydrophilic aliphatic dimethylacrylate (1%–10%), Silanated titanium dioxide ( < 5%) Silanised aluminum oxide filler, TEGDMA ( < 5%), Surface treated aluminum oxide filler (1%–5%), Colloidal silica ( < 0.1%–1%), dl‐Camphorquinone ( < 0.1%), Initiators ( < 1%), Accelerators ( < 2%), Pigments ( < 0.1%)	—
K‐Etchant Syringe Kuraray Noritake Dental Inc., Okayama, Japan		AJ0282	Phosphoric acid 35%, water, colloidal silica, pigment	
Clearfil Majesty ES‐2 Kuraray Noritake Dental Inc, Okayama, Japan		B70025	Bis‐GMA, Silanated barium glass filler, Pre‐polymerized organic filler, Hydrophobic aromatic dimethacrylate, Hydrophobic aliphatic dimethacrylate, dl‐Camphorquinone, Accelerators, Initiators, Pigments	
Calibra Silane Coupling Agent Dentsply Sirona, North Carolina, USA		2306000061	Ethyl Alcohol (92.6%), Acetone (7.4%), γ‐Methacryloxypropyl‐Trimethoxysilane	Apply 30 s, rinse 20 s, air dry
**Universal adhesives**				
Clearfil Universal Bond Quick/Kuraray Noritake Dental Inc., Okayama, Japan	CUBQ	—	10‐MDP, Bis‐GMA, 2‐HEMA, hydrophilic aliphatic dimethacrylate, colloidal silica, silane coupling agent, dl‐camphor‐ quinone, ethanol, water, Sodium fluoride, initiators, accelerators	Apply, massage in for 10 s, air dry for 5 s, light cure for 10 s.
G‐Premio Bond GC Corporation Tokyo, Japan	GPB	2401104	Aceton (25% to < 50%), 2‐Hydroxy‐1,3‐dimethacryloxypropan ( > 10% to < 20%), 10‐MDP (5% to < 10%), silicic acid (5% to < 10%), 2,2'‐Ethylenedioxydiethyldimethacrylat (2.5% to < 5%), Diphenyl(2,4,6‐trimethylbenzoyl) phosphinoxid ( > 0.25% to < 0.5%), 2,6‐Di‐*tert*‐butyl‐*p*‐Cresol, aluminum oxide (0.2% to < 0.5%),	Apply, wait 10 s, dry 5 s, light cure 10 s.
Peak Universal Bond Ultradent Products Inc., South Jordan, UT, USA	PUB	BXKG8	Methacrylic acid ( < 6%), Phenyl‐bis(2,4,6‐trimethylbenzoyl)‐phosphine oxide, ethyl alcohol, 2‐HEMA, Chlorhexidine di(acetate), Dymetech phosphate monomer blend (blend of three phosphate monomers and 12 cross‐linking methacrylate groups).	Apply 10 s, air dry 10 s, light cure 10 s.
Adhese Universal Ivoclar Vivadent, Schaan, Liechtenstein	AU	Z06RST	2‐HEMA, Bis‐GMA, Ethanol, D3MA, Methacrylate phosphoric acid ester, Campherchinon, 2‐Dimethylaminoethylmethacrylat, 10‐MDP, MCAP, highly dispersed silica, water	Apply 20 s, air dry, light cure 10 s.
Scotchbond Universal Plus 3M Deutschland GmbH Neuss, Germany	SBUP	10292078	10‐MDP, Phosphate Monomer, 2‐HEMA, Vitrebond copolymer, silica filler, ethanol, water, initiators, silane, photoinitiator system, dual‐cure accelerator, BPA derivate free dimethacrylate monomers	Apply for 20 s, air dry for 5 s, light cure for 10 s.
Panavia V5; Tooth Primer Kuraray Noritake Dental Inc, Okayama, Japan		3U0276	2‐HEMA, 2‐Dimethylaminoethyl methacrylate, 10‐MDP, Hydrophilic aliphatic dimethacrylate, Accelerator, water	Apply for 20 s, air dry for 5 s

Abbreviations: 10‐MDP, 10‐methacryloyloxydecyl‐dihydrogenphosphat; Bis‐GMA, Bisphenol‐A diglycidylmethacrylate; 2‐HEMA, 2‐hydroxyethylmethacrylat; TEGDMA, triethyleneglycol dimethacrylate; D3MA, 1,10‐decandioldimethacrylat; MCAP, methacrylated carboxylic acid polymer.

### 2.3 Composite Cylinder Preparation

Composite cylinders (5 mm thickness, 2.5 mm diameter) were fabricated using Clearfil Majesty ES‐2 Universal, shade U (Kuraray Noritake, Japan) within a Teflon mold to ensure standardized geometry. The composite material was incrementally applied in three layers (2, 2, and 1 mm thickness) and light‐cured per increment (1200 mW/cm²/20 s) using an LED curing unit (Bluephase Style, Ivoclar Vivadent, Germany) with occlusal (top‐down) irradiation onto the exposed composite surface. Following mold removal, the composite cylinders were further polymerized by light‐curing from the buccal, lingual, mesial, and distal aspects, with each exposure lasting 20 s, ensuring comprehensive polymerization. Subsequently, one randomly selected surface of each composite specimen was standardized by polishing with 600‐grit silicon carbide (SiC) abrasive paper under water cooling, followed by sandblasting with 50 μm aluminum oxide particles (0.2 MPa/10 s) to achieve a uniform bonding surface.

### 2.4 Cementation Process

**Figure 2:**
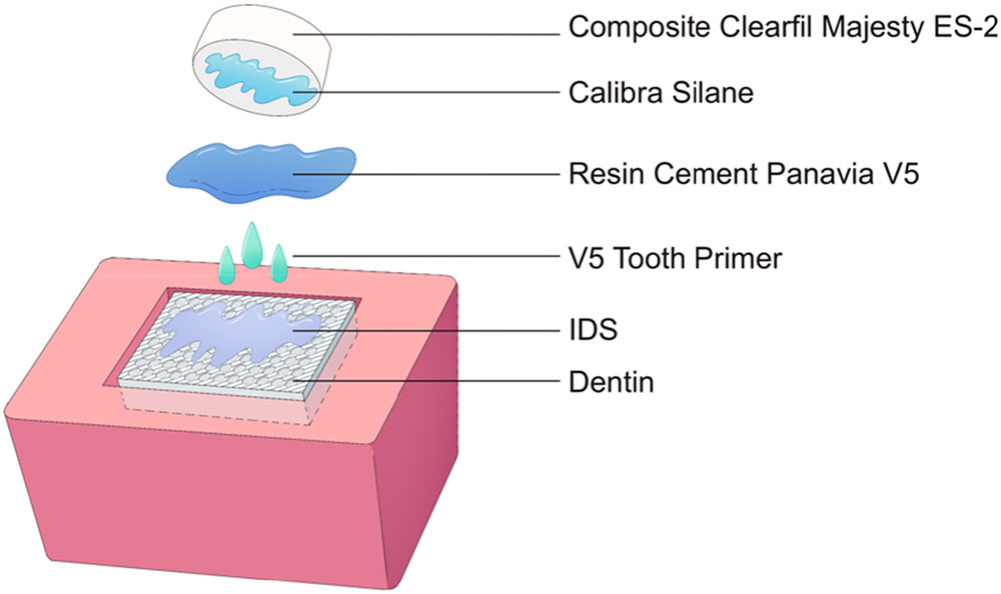
Schematic illustration of the bonding procedure used in the study.

To condition the dentin surfaces before cementation, Panavia V5 Tooth Primer (Kuraray Noritake, Japan) was applied to all dentin specimens in accordance with the manufacturer's instructions (Figure 2). After applying the primer, it was left for 20 s before being gently blown dry to remove excess solvent. For the cementation process, all composite blocks were ultrasonically cleaned in distilled water for 10 min, then dried using compressed air and cotton pellets. To ensure methodological consistency and eliminate potential confounding variables, a uniform silane coupling agent (Calibra Silane, Dentsply Sirona, Bensheim, Germany) was applied across all composite specimens. Composite blocks were cemented using a dual‐cure adhesive resin cement (PAN, Panavia V5, Kuraray Noritake, Japan) and subsequently light‐cured (1200 mW/cm²/40 s) with LED curing unit (Bluephase Style, Ivoclar Vivadent, Ellwangen, Germany). To ensure complete polymerization, the light was initially applied occlusally (top‐down) onto the exposed surface of the cemented rod, followed by additional curing from the buccal, lingual, mesial, and distal directions. This multi‐directional curing approach was implemented to optimize light penetration and compensate for potential shadowing effects. All steps were performed by a single operator (MJ), ensuring consistency across all specimens.

### 2.5 Thermal Aging

Half of the specimens were then randomly assigned to an aged group. These specimens were stored in distilled water at 37°C for 30 days, followed by 10,000 thermocycles between 5°C and 55°C with a dwell time of 30 s per bath and a transfer time of 5 s (RC 20 CS, Lauda, Germany). The remaining specimens comprised the non‐aged group and were stored in distilled water at 37°C for 24 h before testing (Shafiei et al. 2025).

### 2.6 Measurement of SBS

The SBS test was conducted using a universal testing machine (zwickiLine Z0.5 TN, Zwick Roell, Ulm, Germany) at a crosshead speed of 0.5 mm/min. Each specimen was positioned such that the bonded surface was precisely aligned with the force application mechanism. A knife‐edge shear blade was used to apply the load directly at the cement‐rod‐bond resin interface, ensuring consistent and accurate force distribution. The blade was positioned flush against the interface to prevent stress concentration artifacts and ensure a uniform shear force application (Figure S1). The failure load, recorded in Newtons (N), was divided by the bonded surface area (19.63 mm² per specimen) to calculate the SBS (MPa).

### 2.7 Failure Mode and SEM Analysis

After debonding, the fractured specimens were analyzed using a digital microscope (VHX‐5000, Keyence Corp., Osaka, Japan) at a 40× magnification and classified into five distinct failure modes to characterize bond integrity (Deniz et al. 2021):

1. Adhesive failure at the dentin‐adhesive interface – bond failure occurring between the dentin and the adhesive layer.
2. Adhesive failure at the IDS‐cement interface – failure at the interface between the immediate dentin sealing (IDS) layer and the resin cement.
3. Cohesive failure within dentin – fracture propagation through the dentin substrate itself.
4. Cohesive failure within the cement – failure occurring entirely within the cement structure.
5. Mixed failure – a combination of adhesive and cohesive failure patterns.

For scanning electron microscopy (SEM) analysis, a representative sample from each group was randomly selected to examine the failure mode. These specimens were dried and subsequently coated with a gold‐palladium alloy using a sputter coater (Q150T Plus, Quorum, UK). Imaging was performed at 400x and 1000x magnification (1536 × 1024 pixels) utilizing a SEM (Sigma 360 VP, Carl Zeiss, Germany).

### 2.8 Statistical Analysis

Statistical analysis was performed using IBM SPSS Statistics v24 (IBM Corp., Armonk, NY, USA). A Generalized Linear Model (GLM) was applied to evaluate the effects of adhesive type, aging condition (24 h vs. 10.000 thermocycles), and their interaction on SBS. Given the non‐normal distribution of residuals (Shapiro–Wilk test, *p* < 0.001) and the violation of homogeneity of variances (Levene's test, *p* < 0.001), a Gamma distribution with a logarithmic link function was used. Pairwise comparisons for significant main effects were conducted using the Bonferroni correction with a significance level at 5%.

## 3 Results

### 3.1 Shear Bond Strength

Mean SBS values and standard deviations for all groups are presented in Table 2.

**Table 2:** Shear bond strength values (mean ± standard deviation, in MPa) of six self‐adhesive resin cements used, evaluated at two time points: non‐aged and aged.

Universal adhesive	24 h water storage non‐aged	Thermocycling (10.000 × cycles) aged
**NOC**	7.99 ± 0.48 ^bcdef^	5.57 ± 0.53^bcde^
**SBUP**	18.94 ± 2.1^af^	16.36 ± 3.39^aef^
**GPB**	17.32 ± 1.90 ^af^	14.93 ± 3.63^aef^
**AU**	17.28 ± 3.37 ^af^	12.73 ± 4.3^aD^
**CUBQ**	15.51 ± 3.66 ^a^	10.46 ± 3.50^abcE^
**PUB**	12.82 ± 6.06 ^abcd^	9.44 ± 4.02^bc^
Total	14.98 ± 4.95	11.58 ± 5.22

*Note:* Superscript letters (a–f) indicate significant differences between the universal adhesive groups, while capital letters (A–F) represent significant differences within each group across aging conditions (*p* < 0.05).

Abbreviations: AU, Adhese Universal, Ivoclar Vivadent; CUBQ, clearfil universal bond Quick, Kuraray Noritake; GPB, G‐Premio Bond, GC Dental; NOC, negative control, no IDS‐treatment was applied; PUB, peak universal bond, Ultradent Products; SBUP, Scotchbond Universal Plus, 3M, ‘Total’ row represents the overall mean ± SD across all adhesive groups for each aging condition.

Statistical analysis showed a significant effect of thermocycling on SBS (*F* = 43.754, *df* = 1, *p* < 0.001, η² = 0.207). Significant differences were also found among the tested universal adhesives (*F* = 38.977, *df* = 5, *p* < 0.001, η² = 0.537). The interaction between adhesive type and aging condition was not statistically significant (*F* = 0.847, *df* = 5, *p* = 0.518, η² = 0.025).

Pairwise comparisons revealed significant differences in SBS values between adhesives at both time points (24 h and after 10,000 thermocycles). SBUP exhibited the highest bond strength values at both time points (18.94 ± 2.11 MPa at 24 h; 16.36 ± 3.39 MPa after thermocycling). GPB and AU also showed high initial bond strengths, but both adhesives experienced a more pronounced decrease after thermocycling.

Within the IDS group, PUB exhibited the lowest SBS values (12.82 ± 6.06 MPa at 24 h; 9.44 ± 4.02 MPa after thermocycling). In contrast, the negative control (NOC) without IDS treatment demonstrated the overall lowest bond strength (7.99 ± 0.48 MPa at 24 h; 5.57 ± 0.53 MPa after thermocycling). Overall, thermocycling significantly reduced bond strength across all adhesives, with the most pronounced decrease observed for CUBQ (from 15.51 ± 3.66 MPa to 10.46 ± 3.50 MPa) and AU (from 17.28 ± 3.37 MPa to 12.73 ± 4.34 MPa).

### 3.2 Failure Modes

**Figure 3:**
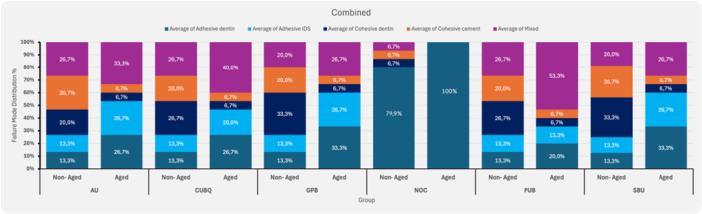
Failure mode distribution after 24 h of water storage and after 30 days of water storage, followed by 10,000 thermocycling cycles for the experimental groups, including five different universal adhesives and a control group. AU, Adhese Universal; CUBQ, Clearfil Universal Bond Quick; GPB, G‐Premio Bond; NOC, Control, no IDS; PUB, Peak Universal Bond; SBUP, Scotchbond Universal Plus.

The distribution of failure modes for all groups is presented in Figure 3. At 24 h, NOC exhibited predominantly adhesive failures at the dentin interface (66.7%), with fewer cohesive failures in dentin (6.7%) and cement (6.7%). Mixed failures were observed in 6.7% of cases. In groups treated with universal adhesives and IDS, failure modes were more evenly distributed. Cohesive failures within dentin were highest in GPB (33.3%), followed by CUBQ, PUB, and AU (20–26.7%). Cohesive cement failures ranged from 20.0% (CUBQ, GPB, PUB) to 26.7% (AU). Mixed failures occurred in 26.7% (AU, CUBQ, PUB) of cases.

After aging (10,000 thermocycles), adhesive failures at the dentin interface increased across all groups, particularly in NOC (93.3%). In IDS‐treated groups, adhesive failures ranged from 20.0% (PUB) to 33.3% (GPB). Mixed failures increased, most notably in PUB (53.3%), CUBQ (40.0%), and AU (33.3%).

### 3.3 SEM

**Figure 4:**
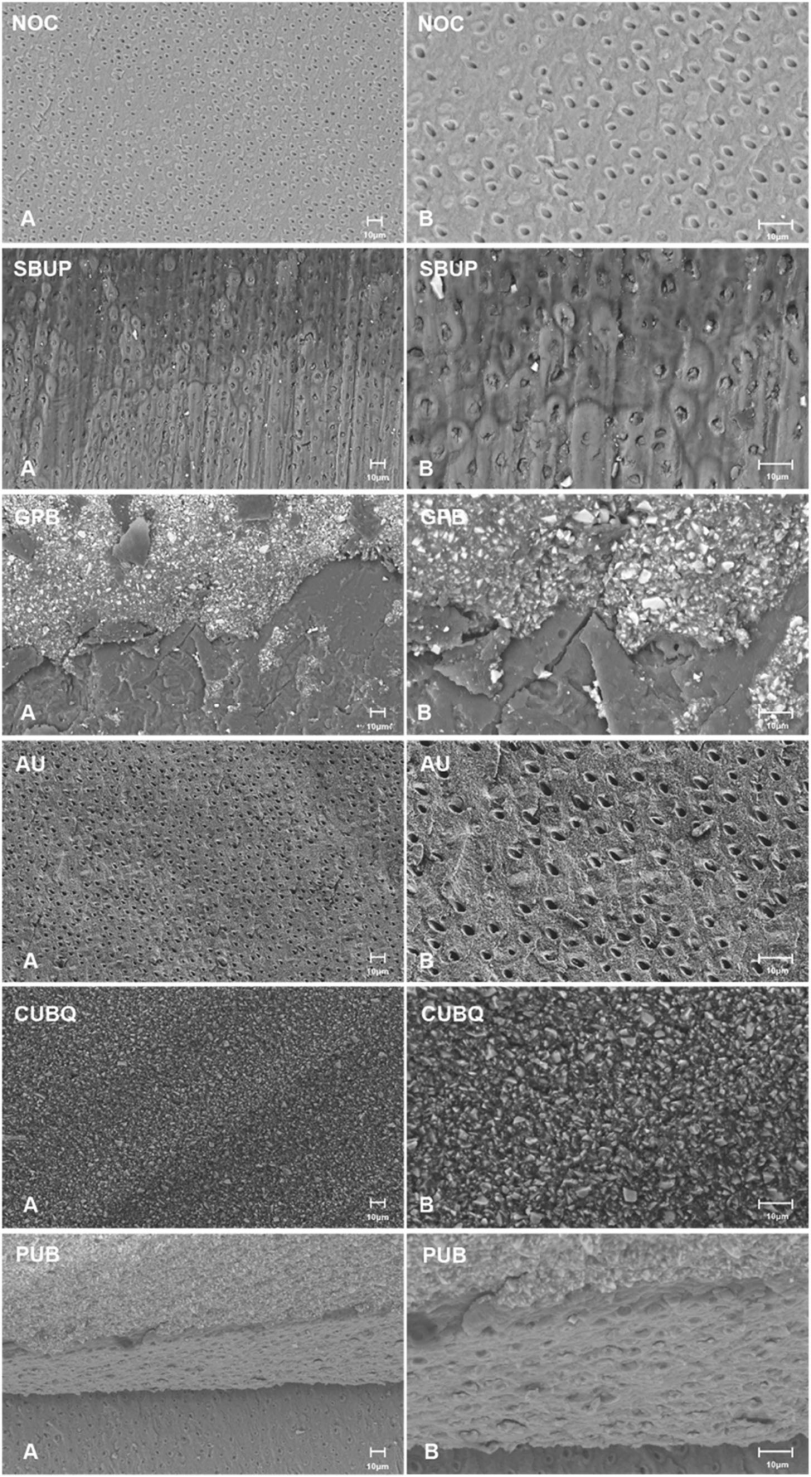
Representative scanning electron microscope images (A) 400×, (B) 1000× magnification of dentin surfaces after 30 days of water storage and 10,000 thermocycles, illustrating the failure modes observed in the different study groups: adhesive failure in dentin (NOC, SBUP), adhesive failure at the IDS‐cement interface (GPB), cohesive failure in dentin (AU), cohesive failure within the cement (CUBQ), and mixed failure (PUB). AU, Adhese Universal; CUBQ, Clearfil Universal Bond Quick; GPB, G‐Premio Bond; NOC, Control (No IDS); PUB, Peak Universal Bond; SBUP, Scotchbond Universal Plus.

Representative SEM images of the dentin surfaces after 10,000 thermocycles are shown in Figure 4. In NOC and SBUP groups, adhesive failure in dentin was predominantly observed, resulting in exposed dentin surfaces with minimal residual adhesive. In the GPB group, adhesive failure at the IDS‐cement interface was prevalent, characterized by a thin, disrupted adhesive layer with visible resin remnants. Cohesive failure within the dentin, as observed in the AU group, exhibited irregular fracture propagation through the dentin matrix. In contrast, cohesive failure within the cement, as seen in the CUBQ group, resulted in a rough, fragmented cement surface. Mixed failures presented a combination of adhesive debonding and cohesive disruption, frequently showing residual cement remnants on the dentin surface.

## 4 Discussion

This study investigated the influence of the Immediate Dentin Sealing technique on the SBS of dual‐cure luting cement using various universal adhesives, both immediately after cementation and following thermal aging. The results demonstrated that IDS significantly enhanced bond strength, particularly with SBUP and GPB. After thermal aging, these adhesives exhibited greater stability, whereas CUBQ and AU showed the most pronounced reductions in SBS. The NOC group, which lacked IDS, displayed the lowest bond strength at both time points. IDS also influenced failure modes, with cohesive and mixed failures predominating in IDS‐treated groups, whereas adhesive failures were more frequent in specimens without IDS. Based on these findings, the null hypotheses were rejected, as significant differences were identified both among the tested universal adhesives and between the IDS technique and conventional cementation.

The performance differences among UAs within the IDS protocol are primarily attributed to their chemical composition. Universal adhesives typically consist of functional monomers, solvents, and photoinitiators, whose interplay significantly influences adhesive quality. Notably, the adhesive performance of MDP‐based systems extends beyond dentin, as these monomers are also used for durable composite repairs on zirconia due to their affinity to metal oxides (Janson et al. 2025). In this study, MDP‐containing adhesives, such as SBUP and GPB, exhibited the highest SBS values, which aligns with previous findings on the chemical interaction of 10‐methacryloyloxydecyl dihydrogen phosphate (10‐MDP) with the calcium phase of hydroxyapatite crystals (Antoniou et al. 2024; Fazlioglu et al. 2025). In contrast, methacrylate phosphoric acid esters (AU, PUB) showed weaker bonding potential, likely due to reduced interaction with dentin. Despite containing 10‐MDP, CUBQ demonstrated lower SBS, possibly due to the viscosity‐enhancing effects of silica, which may impair adhesive homogeneity. PUB, lacking 10‐MDP, relied on methacrylic acid and HEMA, which provide limited chemical bonding and may restrict smear layer removal due to its higher pH.

The solvent system plays a crucial role in adhesive stability. Acetone‐based adhesives (e.g., GPB) promote deep resin penetration but are highly volatile, making them prone to phase separation if not applied correctly or exposed to air for extended periods (Kimyai et al. 2023) This instability can result in an inhomogeneous adhesive layer, reducing bond strength. Following thermal aging, these adhesives may weaken further due to incomplete resin infiltration, as rapid solvent evaporation can compromise interfacial integrity (Schoppmeier et al. 2025). Ethanol‐based systems (e.g., AU) allow for controlled monomer diffusion, theoretically improving polymerization, yet AU exhibited a notable SBS reduction after aging, suggesting that hydrolytic degradation plays a more critical role than solvent type alone.

Despite these chemical factors, additional environmental influences—such as thermocycling—pose further challenges to bond durability. Thermocycling led to a general SBS reduction across all groups, in line with prior studies highlighting that thermal stress and hydrolysis contribute to interfacial degradation (Shafiei et al. 2025). Temperature fluctuations accelerate resin breakdown, particularly in adhesives with high water absorption. Despite this decline, SBUP, GPB, and AU retained higher bond strengths post‐aging, indicating superior polymer stability. SBUP benefits from the presence of 10‐MDP, HEMA, and a Vitrebond copolymer, while GPB's high acetone content enhances adhesive infiltration—likely due to rapid solvent evaporation during drying. Although acetone‐based adhesives have previously been associated with reduced long‐term stability, the sustained bond strength observed after 10,000 thermocycles in this study contradicts such concerns for GPB, suggesting that its stability under aging conditions may be more favorable than previously assumed.

These findings are consistent with and further substantiated by emerging evidence on the efficacy of the IDS technique in enhancing adhesive performance and mitigating aging‐induced degradation. It has been reported that, among various universal adhesives tested, Scotchbond Universal exhibited the highest microtensile bond strength following thermocycling, while Clearfil Universal Bond Quick showed the most pronounced reduction—an outcome that closely parallels the present findings (Fazlioglu et al. 2025). Notably, their study also highlighted the presence of a robust hybrid layer and long resin tags in high‐performing adhesives, which are key microstructural determinants of interfacial durability. The predominance of mixed and cohesive failure modes observed in our IDS groups further aligns with their SEM findings, reinforcing the interpretation that immediate hybrid layer formation enhances mechanical interlocking and resistance to hydrolytic breakdown (Fazlioglu et al. 2025).

Similarly, previous findings demonstrated that immediate dentin sealing significantly improved bond strength compared to delayed dentin sealing, irrespective of the adhesive application mode, and maintained interfacial integrity even after 10,000 thermocycles (Kimyai et al. 2023). This supports the hypothesis that early sealing of freshly cut dentin not only optimizes initial micromechanical retention but also enhances resistance to thermomechanical stress over time (Tahoun et al. 2024). Furthermore, the data indicated that universal adhesives applied in the etch‐and‐rinse mode maintained their bond strength over a 1‐year period, whereas adhesives used in the self‐etch mode exhibited a significant decline (Shafiei et al. 2025). Their findings underscore the importance of the etching strategy, particularly for adhesives with mild acidity or limited chemical reactivity.

Collectively, these studies converge in demonstrating that the IDS technique confers a significant advantage in both immediate and aged bonding performance—particularly when paired with adhesives featuring stable monomer formulations and favorable solvent systems. The superior post‐aging performance of SBUP and GPB observed in our study not only aligns with these previous findings but also reinforces the clinical relevance of selecting universal adhesives that facilitate effective resin infiltration, hybrid layer stability, and chemical interaction with dentin. These parameters appear critical to sustaining adhesion under thermomechanical stress and should inform future recommendations for IDS‐based bonding protocols.

In contrast, CUBQ and PUB exhibited the weakest post‐aging performance, indicating distinct failure mechanisms. The lower bond strength observed for CUBQ may stem from viscosity‐related limitations in adhesive infiltration, while PUB's higher pH may have reduced its interaction with the demineralized dentin matrix, thereby limiting micromechanical retention and compromising long‐term interfacial stability. AU's monomer balance, by contrast, likely optimized polymerization efficiency and contributed to its relatively better performance. These results emphasize that, beyond chemical composition, both application technique and polymerization quality are crucial for long‐term bonding success. Selecting adhesives with enhanced hydrolytic resistance and improved polymer network formation remains central to optimizing the durability of IDS protocols.

Beyond adhesive selection, the choice of resin cement is a critical factor influencing the overall bonding performance (Yücel et al. 2025). PAN, with its dual‐curing mechanism, ensures complete polymerization even in areas with limited light exposure, enhancing mechanical strength and cross‐linking within the cement matrix. Its hydroperoxide‐thiourea catalyst system, activated by vanadium ions in V5 Tooth Primer, stabilizes polymerization and ensures uniform monomer conversion. Since PAN lacks adhesive monomers, bonding relies entirely on the primer's chemical interaction with dentin. The combination of self‐curing activation and controlled light exposure optimizes polymerization in less accessible regions, improving adhesion and long‐term stability. The curing protocol used in this study minimized incomplete polymerization, reinforcing the durability of the adhesive interface

PAN demonstrated bond strength values aligning with reported thresholds for reliable adhesion. Literature suggests that an SBS of approximately 5 MPa is necessary for long‐term stability of adhesive restorations (Küçükekenci et al. 2024). While these values are appropriate under in vitro conditions, their direct clinical relevance requires further validation. These findings highlight that bond durability depends on monomer composition, solvent properties, and polymerization behavior. While 10‐MDP‐based adhesives provide strong initial adhesion, long‐term stability is dictated by solvent composition and polymer cross‐linking efficiency. The prevalence of adhesive failures after aging underscores the need for optimizing adhesive formulations to improve hydrolytic and mechanical resistance.

High bond strength does not necessarily indicate improved failure resistance. SBU and GPB predominantly exhibited adhesive failures after aging, suggesting that their adhesive interfaces were the weakest link, likely due to solvent volatility or hydrolytic degradation. PUB showed more mixed failures, indicating balanced adhesion possibly due to methacrylic acid's demineralization effect, which enhancing resin infiltration without excessive dentin weakening. AU and CUBQ exhibited both adhesive and cohesive failures, reflecting reliance on chemical interaction and micromechanical retention but with lower aging resistance than SBU and GPB. In the NOC group, adhesive failures predominantly exposed dentin surfaces with minimal adhesive remnants, indicating suboptimal bonding. Mixed failures in the NOC and SBUP groups can be attributed to monomer composition, particularly 10‐MDP, HEMA, and a Vitrebond copolymer, promoting strong chemical bonding. These interfacial failures suggest that bond strength at the adhesive‐dentin interface surpassed the adhesive layer's cohesive strength under thermomechanical stress, causing partial debonding rather than cohesive failure. This aligns with previous findings that adhesive and mixed failures reflect stress distribution and material dynamics under load rather than weaker adhesion (Samimi et al. 2024; Yang et al. 2024; Pascoe 2023). Similarly, disrupted adhesive layers and resin residues in GPB highlighted interfacial weaknesses. In contrast, cohesive failures within dentin (AU) and cement (CUBQ) resulted from localized stress accumulation rather than inherently stronger adhesion. The prevalence of mixed failures underscores the limitations of current bonding strategies, emphasizing the need for optimized surface pretreatment to improve long‐term adhesion. Cohesive fractures, while observed in certain cases, should not be directly interpreted as an indicator of bond strength between the adhesive and resin cement. Instead, failure patterns are primarily influenced by the stochastic nature of crack propagation, which is governed by intrinsic material defects, localized stress distribution, and the direction of crack initiation and propagation (Pascoe 2023). Even well‐polymerized resin cement can exhibit cohesive failures due to internal weaknesses. Similarly, adhesive and mixed failures often result from interfacial defects rather than bond strength variability.

### 4.1 Limitations

Despite its valuable insights, this study has limitations. The 10,000‐cycle thermocycling protocol approximates 1 year of aging but may not fully reflect long‐term clinical conditions. Extended aging methods, such as higher cycle counts or prolonged water storage, could provide a more comprehensive assessment. Additionally, the in vitro design does not replicate masticatory forces, pH fluctuations, or enzymatic degradation, all of which affect bond longevity. The use of a single silane coupling agent (Calibra Silane, Dentsply Sirona, Germany) for all adhesives may have influenced results, as manufacturer‐specific silanes vary in composition and compatibility. Furthermore, universal adhesives were tested exclusively in etch‐and‐rinse mode, which could have impacted outcomes given the mode‐dependent performance of these adhesives. Lastly, the knife‐edge indenter in SBS testing may have increased stress concentration at the bonded interface compared to the recommended notched‐end design. Future studies should consider the latter to minimize stress artifacts and enhance result standardization.

## 5 Conclusion

The application of UAs as IDS agents significantly enhances bond strength to dentin compared to conventional cementation without IDS. However, thermocycling led to a reduction in bond strength across all groups, highlighting the impact of aging on adhesive durability. Among the tested adhesives, SBUP and GPB demonstrated the highest bond strength both initially and after aging, making them the most clinically favorable options. Their superior performance is likely attributable to monomer formulations containing 10‐MDP and acetone, which enhance chemical interaction with hydroxyapatite and facilitate deep resin infiltration into the dentin substrate. These findings emphasize the clinical relevance of IDS and the importance of selecting UAs with stable adhesive properties to optimize bonding protocols and improve the longevity of indirect restorations.

## Author Contributions

**Conceptualization:** Malin Janson and Anja Liebermann. **Methodology:** Malin Janson, Anja Liebermann, and Christoph Matthias Schoppmeier. **Validation:** Malin Janson and Christoph Matthias Schoppmeier. **Visualization:** Li Sun. **Writing – original draft preparation:** Malin Janson, Anja Liebermann, and Christoph Matthias Schoppmeier. **Review and editing:** Malin Janson, Christoph Matthias Schoppmeier, and Li Sun. **Project administration:** Christoph Schoppmeier and Malin Janson. **Supervision:** Anja Liebermann.

## Ethics Statement

The authors have nothing to report.

## Consent

The authors have nothing to report.

## Conflicts of Interest

The authors declare no conflicts of interest.

## Supporting information

supmat.

## Data Availability

The data that support the findings of this study are available from the corresponding author upon reasonable request.
