# Supplementary material for: Impact of Immediate Dentin Sealing With Various Universal Adhesives on Shear Bond Strength of Dual‐Cure Resin Cement
Source: Clin Exp Dent Res. 2025 Aug 12;11(4):e70186. doi: 10.1002/cre2.70186 (PMC12340710; doi:10.1002/cre2.70186)
Supplement: Supplementary file 1 — supmat. [file CRE2-11-e70186-s001.docx]

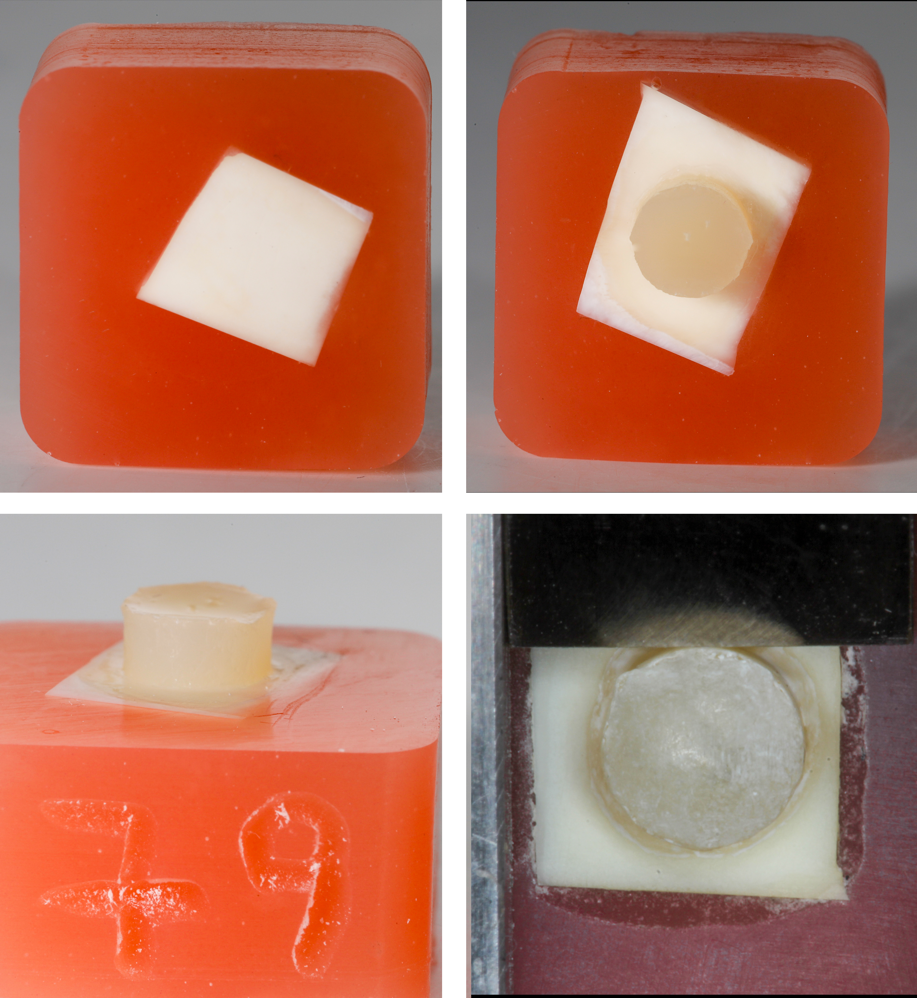


Figure S1 (supplementary material)

Bovine dentin test specimen embedded in resin, with composite resin, mounted in a universal testing machine for shear bond strength testing.
